# Three-dimensional and thermal surface imaging produces reliable measures of joint shape and temperature: a potential tool for quantifying arthritis

**DOI:** 10.1186/ar2360

**Published:** 2008-01-23

**Authors:** Steven J Spalding, C Kent Kwoh, Robert Boudreau, Joseph Enama, Julie Lunich, Daniel Huber, Louis Denes, Raphael Hirsch

**Affiliations:** 1 Division of Rheumatology, Children's Hospital of Pittsburgh, 3705 Fifth Avenue, University of Pittsburgh School of Medicine, Pittsburgh, PA 15213, USA; 2 Department of Medicine, University of Pittsburgh School of Medicine, 3550 Terrace Street, Pittsburgh, PA 15213, USA; 3 Robotics Institute, Carnegie Mellon University, 5000 Forbes Avenue, Pittsburgh, PA 15213, USA

## Abstract

**Introduction:**

The assessment of joints with active arthritis is a core component of widely used outcome measures.  However, substantial variability exists within and across examiners in assessment of these active joint counts.  Swelling  and  temperature changes, two qualities estimated during active joint counts, are amenable to quantification using noncontact digital  imaging  technologies.  We  sought to explore the  ability of three dimensional (3D) and thermal imaging to reliably measure joint shape and temperature.

**Methods:**

A Minolta 910 Vivid non-contact 3D laser scanner and a Meditherm med2000  Pro Infrared camera were used to create digital representations of wrist and metacarpalphalangeal (MCP) joints.  Specialized software generated 3 quantitative measures for each joint region: 1) Volume; 2) Surface Distribution Index (SDI), a marker of joint shape representing the standard deviation of vertical distances from points on the skin surface to a fixed reference plane; 3) Heat Distribution Index (HDI), representing the standard error of temperatures.  Seven wrists and 6 MCP  regions from 5 subjects with arthritis were used to develop and validate 3D image acquisition and processing techniques.  HDI values from 18 wrist and 9 MCP regions were obtained from 17 patients with active arthritis and compared to data from 10 wrist and MCP regions from 5 controls.  Standard deviation (SD), coefficient of variation (CV), and intraclass correlation coefficients (ICC) were calculated for each quantitative measure to establish  their reliability.  CVs for volume and SDI were <1.3% and ICCs were greater than 0.99.

**Results:**

Thermal measures were less reliable than 3D measures.  However, significant differences were observed between control and arthritis HDI values.  Two case studies of  arthritic joints demonstrated quantifiable changes in swelling and temperature corresponding with changes in symptoms and physical exam findings.

**Conclusion:**

3D and thermal imaging provide reliable measures of joint volume, shape, and thermal patterns.  Further refinement may lead to the use of these technologies to improve the assessment of disease activity in arthritis.

## Introduction

Rheumatoid arthritis (RA) and juvenile idiopathic arthritis (JIA) are chronic inflammatory conditions of the joints which can result in substantial morbidity and loss of function. Over the last decade, significant progress has been made in increasing the number pharmacological options available to treat these conditions. To determine the efficacy of these new drug therapies, outcome measures, such as the American College of Rheumatology (ACR) 20 in RA and the ACR 30 in JIA, have been developed and accepted by international regulatory agencies [1,2]. An essential component of these outcome measures is the assessment of the number of joints with active arthritis. Unfortunately, carefully designed studies have repeatedly shown poor reproducibility of physician-assessed swollen joint counts and active joint counts. Studies of intra- and inter-observer variability regarding these measures have demonstrated high coefficients of variation (CVs) and low intra-class correlation coefficients (ICCs) [3-5]. An unbiased and reliable measure of the inflammatory state of the joint would improve the ability to quantify disease activity. Such a measure could be used to assess response to therapy in both the clinical and research settings.

A number of imaging technologies have been studied in an effort to improve the assessment of arthritis activity. However, all of the current technologies have limitations. For instance, plain radiographs are insensitive to early changes. Ultrasound can quantify changes in effusion and synovitis, but it is highly user-dependent. Magnetic resonance imaging (MRI) has proven to be more sensitive and reliable than clinical examination in the detection of synovitis and has the ability to quantify changes in synovial volumes and erosions [3,6]. However, MRI involves substantial time and cost, exposure to contrast agents, and the need for sedation in young children. We conducted a proof-of-concept study to determine whether two of the cardinal signs of disease activity in arthritis (swelling and warmth) can be reliably quantified using existing three-dimensional (3D) and thermal digital imaging devices.

## Materials and methods

### Patients

Seven wrist and 6 metacarpalphalangeal (MCP) regions from 5 subjects with arthritis were used to develop and validate 3D image acquisition and processing techniques. HDI values from 18 wrist and 9 MCP regions were obtained from 17 patients with active arthritis and compared with data from 10 wrist and MCP regions from 5 controls. The subjects included pediatric patients recruited from a single pediatric rheumatology practice and adult patients recruited from an academic rheumatology center. Diagnosis and classification of RA or JIA were made based on accepted ACR criteria or International League Against Rheumatism criteria [7,8]. Active arthritis was defined as the presence of swelling and tenderness. The study protocol was approved by the University of Pittsburgh Institutional Review Board. All patients signed informed consent forms prior to inclusion in the study.

### 3D data acquisition and processing

**Figure 1:**
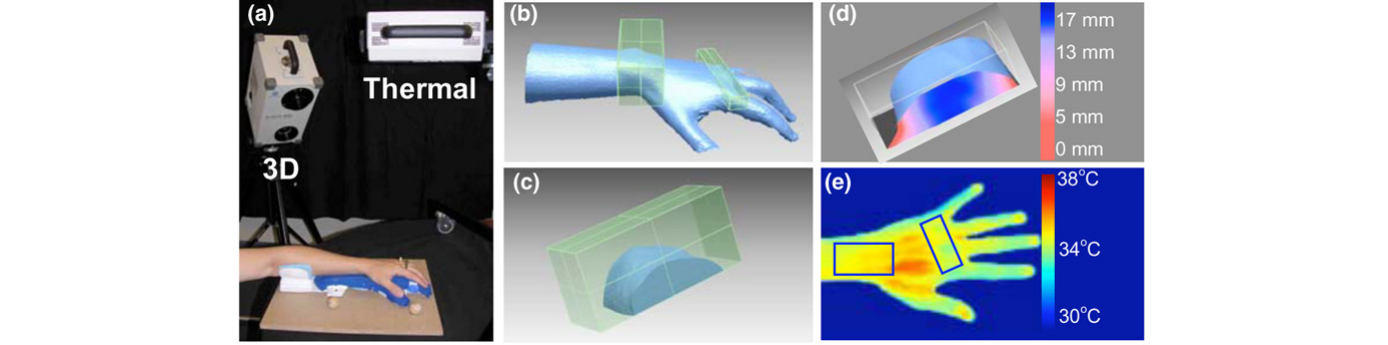
Image acquisition and processing. After immobilizing a subject's wrist and hand in a fixation splint **(a)**, two scans are obtained from opposite view points and the scans are merged to create a three-dimensional (3D) model **(b)**. Using the model, the center of a predefined region of interest (ROI) is selected and defined by the green box. Both wrist and metacarpalphalangeal (MCP) ROI boxes are shown. The ROIs can be isolated and the volume between the base of the ROI and the surface can be directly calculated using Rapidform software. The wrist is shown as an example **(c)**. The distance in millimeters from the base of the ROI to the surface can be depicted as a color map in which blue represents a greater, and red a lesser, distance in millimeters from the base **(d)**. In a similar manner, ROIs defining the wrist and MCP are selected from thermograms **(e) **and used to calculate the heat distribution index.

The image acquisition and processing technique is outlined in Figure 1. A forearm-based hand splint was designed to minimize movement and standardize hand position and pose between sessions (Figure 1a). Fixed objects, necessary to create and align 3D models from different sessions, were attached to the base of this splint. Scans were acquired using a Minolta Vivid 910 (Konica Minolta Sensing Americas, Inc., Ramsey, NJ, USA), a laser line triangulation scanner that produces a 640 × 480-pixel 3D image. Its manufacturer-reported resolution and accuracy are less than 0.2 mm in all axes. The camera was operated via a laptop computer using Polygon Editing Tool (version 1.22; Konica Minolta Sensing Americas, Inc.). Scans were acquired under the following standard conditions: camera positioned perpendicular to the subject's forearm at a stand-off distance of 0.8 m, camera height of 0.8 m, and camera declined to 45° from horizontal. Ambient room lighting was used during image acquisition. Two scans of each hand/wrist were acquired, one from the medial and the other from the lateral side. Rapidform2006 software (INUS Technology, Inc., Seoul, South Korea) was used to merge these two scans into a single, complete 3D model (Figure 1b). To follow patients longitudinally, it was essential to prevent minor wrist or hand rotation from session to session which might cause false variations in volume measurements. The fixation device constructed for this study prevented most such rotation. Small positioning changes that did occur were readily overcome by aligning the forearm and hand of models created across sessions, using the Rapidform co-registration function. Subsequent 3D models were constructed in the same fashion and then aligned to the reference model using the fiduciary markers and stable anatomic landmarks.

After model creation, two distinct computer-generated regions of interest (ROIs) were defined, one for the wrist and one for the 2nd-5th MCPs (green boxes in Figure 1b). The 2nd-5th MCP region was treated as a single ROI because the MCP joints are in juxtaposition to each other, and, in the case in which an MCP is swollen, it is impossible to determine where one MCP region ends and the adjacent one begins. The center of the wrist ROI was defined as the midpoint of the distance between the radial and ulnar styloids, whereas the center of the 2nd-5th MCP ROI was defined as the midpoint of the distance between the peaks of the 3rd and 4th MCP. Through trial and error, we determined that wrist ROI box dimensions of 9 cm in the medial-lateral plane, 4 cm in the proximal-distal plane, and 4 cm in the vertical plane and MCP ROI box dimensions of 10 cm in the medial-lateral plane, 2 cm in the proximal-distal plane, and 2 cm in the vertical plane encompassed maximal relevant data. These ROI boxes were created at the initial imaging session and remained fixed for all subsequent sessions. The wrist or MCP ROI was then extracted by deleting all data outside of the ROI box (Figure 1c). Volumes within the ROIs were then calculated. In addition, all points on the joint surface could be represented as distances in millimeters from the bottom plane of the ROI box. These distances could then be depicted as a color map (Figure 1d). We have established a surface distribution index (SDI), defined as one standard deviation (SD) from the mean of the all surface points-to-bottom plane distances. The SDI is a reflection of the surface shape, and distortions due to swelling will result in a change in SDI. The SDI data were generated using the 'Shell-Surface deviation' function in the Rapidform software.

### Thermal data acquisition and processing

Thermal data were acquired using a Meditherm medPro2000 thermoelectrically cooled microbolometer (Meditherm, Inc., Beaufort, NC, USA) and WinTES Thermal Evaluation Software (Compix, Lake Oswego, OR, Queensland, Australia). Unlike other commercially available thermal imagers, this sensor is specifically designed to measure temperatures found in the human body (10°C to 40°C). The device has a manufacturer-reported sensitivity and accuracy of less than 0.1°C and self-calibrates to an internal source at each pixel, avoiding the need for an external calibration target. Following International Academy of Clinical Thermology guidelines [9], subjects were asked to remove all jewelry and clothing covering the joints of interest and were given a 15-minute acclimation period prior to thermal imaging. All thermal images were obtained with the camera positioned directly over the hands. Ambient room temperature was 22°C ± 0.5°C. Skin emissivity was fixed at 0.98 [10,11]. Thermal data were processed using specially designed code in Matlab (The MathWorks, Inc., Natick, MA, USA). With this code, centers for standard ROI boxes were selected (Figure 1e). The midpoint of the wrist or the midpoint between the 3rd and 4th MCPs served as the center of the thermal ROI boxes. A heat distribution index (HDI) was defined as twice the SD of all temperatures within the ROI [8]. Relative frequency distributions were generated by plotting the frequency of temperatures in 1°C increments.

### Statistical analysis

Wrist and MCP volume and shape vary across individuals. Therefore, pooled SDs were used to represent the overall measurement error SD when measuring volume and shape on multiple individuals. Excel XP (Microsoft Corporation, Redmond, WA, USA) and SAS 9.1 (SAS Institute Inc., Cary, NC, USA) were used for analysis. The average CV was used as a measure of overall CV. The ICC [1,3] was used as a measure of reliability [12]. When comparing HDIs, group means were used to examine for significant differences using Student *t *tests. *P *values of less than 0.05 were considered significant. The area under the receiver operating characteristic (ROC) curve was used to assess overall sensitivity and specificity of thermal imaging [13].

## Results

### 3D measures are highly reliable

We tested the reliability of the 3D measures of wrists and MCPs in subjects with arthritis in a clinically relevant setting. To compare inter-session reliability, 7 wrist and 6 MCP regions from 5 subjects (3 JIA and 2 RA) were scanned twice by an experienced camera operator. The subject left the room between each of the imaging sessions. Wrist and MCP volume and SDI measures demonstrated excellent reliability across imaging sessions (Table 1). In wrists, pooled inter-session volume SD was 0.9 ml (CV, 1.3%) and SDI SD was 0.1 mm (CV, 1.1%). Inter-session pooled MCP volume SD was 0.1 ml (CV, 1.3%) and SDI SD was 0.1 mm (CV, 1.1%). ICCs [1,3] for all 3D measures of wrists and MCPs were greater than 0.99 (wrist volume ICC = 0.992, wrist SDI ICC = 0.996, MCP volume ICC = 0.995, and MCP SDI ICC = 0.999). Based on the inter-session data, volume changes greater than 1.1 ml in the wrist and 0.5 ml in the MCPs between imaging sessions would be considered significant with 99% confidence. Similarly, a change in the SDI of 0.4 mm in the wrist or 0.3 mm in the MCPs between imaging sessions would also be significant with the same degree of confidence.

**Table 1:** Reproducibility of wrist and metacarpalphalangeal three-dimensional measures across sessions

3D measure	Wrist							Metacarpophalangeal					
	
	1	2	3	4	5	6	7	1	2	3	4	5	6
Volume, ml													
Mean, ml	55.8	47.8	38.5	39.2	55.0	53.3	66.8	16.7	4.3	8.9	4.0	9.5	7.3
SD, ml	0.7	0.6	0.2	0.2	0.6	2.1	0.5	0.2	0.02	0.1	0.1	0.1	0.1
CV (%)	1.3	1.2	0.6	0.4	1.0	4.0	0.8	1.3	0.4	1.5	1.3	0.9	2.0
SDI, mm													
Mean, mm	10.3	9.3	7.3	7.5	9.0	9.8	10.8	6.7	4.2	5.1	4.5	5.4	4.9
SD, mm	0.2	0.1	0.1	0.03	0.2	0.1	0.02	0.1	0.04	0.1	0.03	0.05	0.04
CV (%)	2.1	1.0	1.2	0.4	1.8	0.7	0.2	1.9	1.0	1.0	0.7	0.9	0.8

3D, three-dimensional; CV, coefficient of variation; SD, standard deviation; SDI, surface distribution index.

### 3D imaging can reliably quantify small changes in joint volume and shape

**Figure 2:**
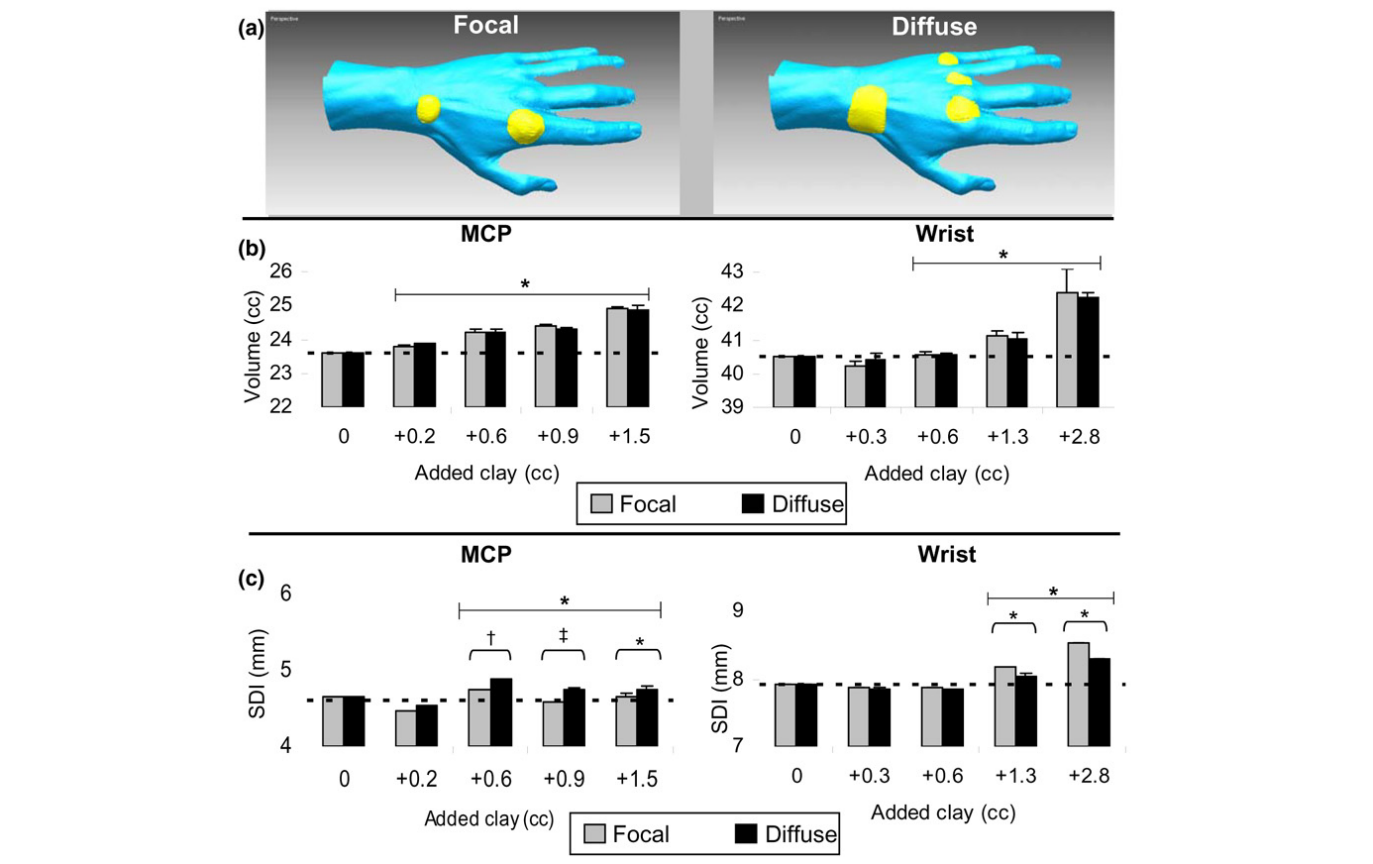
Sensitivity of three-dimensional measures to change due to simulated swelling. Various amount of clay (depicted in yellow) were added to a mannequin wrist and 2nd-5th metacarpalphalangeal (MCP) regions to represent swelling **(a)**. The clay volume was estimated by forming the clay into a cube and measuring the length, width, and height with calipers. Different shapes of the same volume were used to simulate focal and diffuse swelling. Volume changes **(b) **and surface distribution index (SDI) changes **(c) **due to addition of clay are shown, with vertical bars representing the mean and standard deviation of three models. The dotted lines correspond to baseline volume and SDI. Large brackets encompass all values significantly greater than baseline. Small brackets represent comparison of focal and diffuse swelling measurements. *p < 0.05; ^†^*p *< 0.01; ^‡^*p *< 0.001.

We performed a set of experiments to determine the ability of the 3D imager to detect surface changes due to joint swelling, using clay on a mannequin hand to simulate different degrees of swelling consistent with JIA or RA (Figure 2). A known volume of clay was applied to the wrist or MCP region either in a lump, to simulate focal swelling, or spread over a large area, to simulate diffuse swelling (Figure 2a). The estimated changes in volume and SDI were based on the average of three models with the mannequin hand held in fixed position. Three-dimensional imaging proved accurate and sensitive in identifying small changes in both volume and SDI (Figure 2b,c). A significant increase from baseline volume was detectable with the addition of as little as 0.2 ml of clay (0.8% above baseline volume) to the MCP ROI (*p *= 0.0001) and 0.6 ml (1.5% above baseline volume) to the wrist ROI (*p *= 0.0002). A significant increase in SDI from baseline due to simulated swelling was also detected with the addition of as little as 1.3 ml of clay (3.2% above baseline volume) to the wrist ROI (*p *= 0.001) and 0.6 ml (2.5% above baseline volume) added to the MCP ROI (*p *= 0.002). The SDI was able to discriminate between focal and diffuse swelling when 1.6 ml of clay was added to the wrist ROI (*p *= 0.02) and 0.6 ml to the MCP ROI (*p *= 0.0003).

### Thermal imaging differentiates patients with active arthritis from normal controls

**Figure 3:**
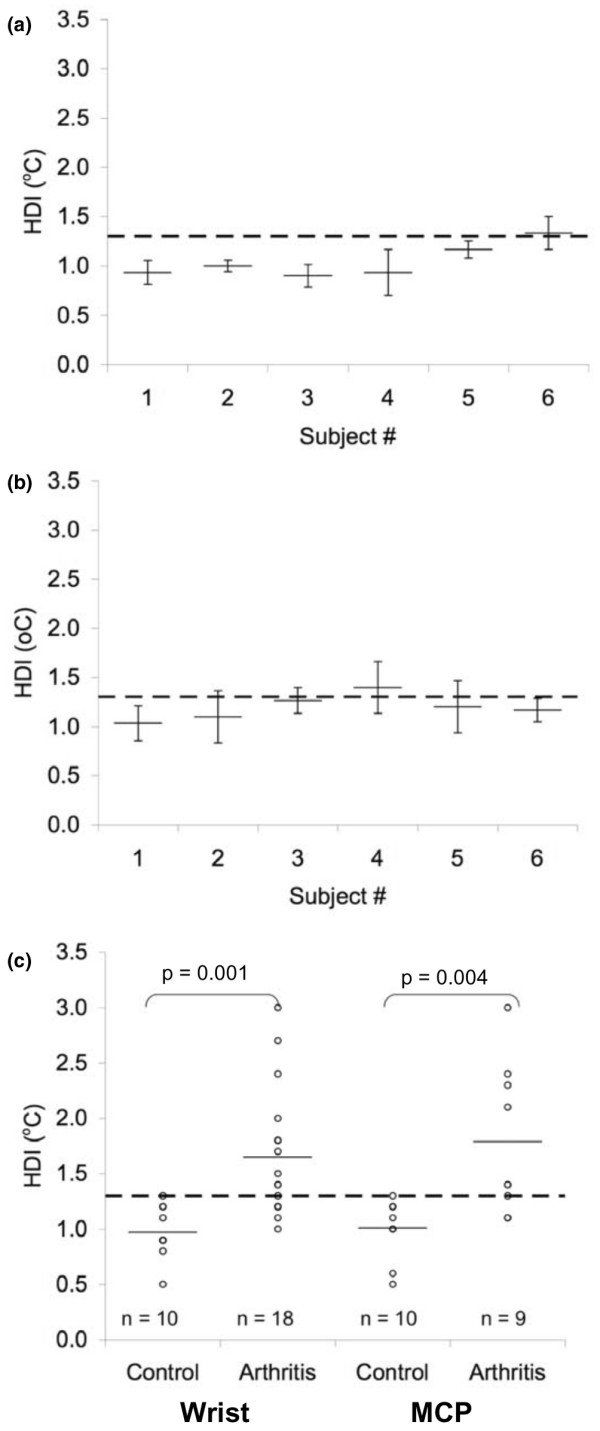
Reproducibility of inter-session human subject wrist **(a) **and metacarpalphalangeal (MCP) **(b) **heat distribution index (HDI) measurements. Three thermal images of control human wrists and MCPs were captured once a day on 3 separate days. Each data point represents the mean and standard error of the three images. **(c) **Comparison of wrist and MCP HDI values in control patients and patients with active arthritis. Solid horizontal lines represent the mean. Dotted line represents proposed cutoff for active arthritis (1.3°C).

To determine the reliability of thermal imaging of the wrist and MCP, 6 normal adult wrists and hands from 3 controls were imaged on 3 separate days. Three thermal scans were obtained at each session and the HDI was calculated for each ROI. Intra-session (that is, same day and time) HDIs were very similar, with SDs less than 0.05°C (data not shown). Pooled inter-session (that is, day-to-day) SD of wrist HDIs was 0.2°C (Figure 3a) whereas MCP HDI performed less well, with an inter-session SD of 0.4°C (Figure 3b). Pooled inter-session CVs were 22.1% for the wrist and 29.7% for the MCP, indicating relatively large day-to-day variation. This was also reflected in the low HDI ICC [1,3] values for wrists (0.146) and MCPs (-0.295). However, no control wrist or MCP HDI exceeded 1.3°C, suggesting that an HDI above 1.3°C might be indicative of the presence of arthritis. To explore this further, we compared HDI values of 10 control wrists and 10 control MCPs to 18 wrists with active arthritis and 9 MCPs with active arthritis. As shown in Figure 3c, an HDI cutoff of 1.3°C discriminated well between controls and patients with active arthritis. The mean ± SD HDI in control joints was 1.0°C ± 0.2°C compared with 1.7°C ± 0.6°C in joints with active arthritis (*p *< 0.0001). By ROC analysis, an HDI value of 1.3°C yielded a sensitivity of 67% and a specificity of 100%. The area under the ROC curve was 0.823. No significant differences in HDI were seen between control adults and children or between arthritic adults and children.

### 3D and thermal surface imaging can quantify clinically meaningful changes in arthritic joints in response to therapy

**Figure 4:**
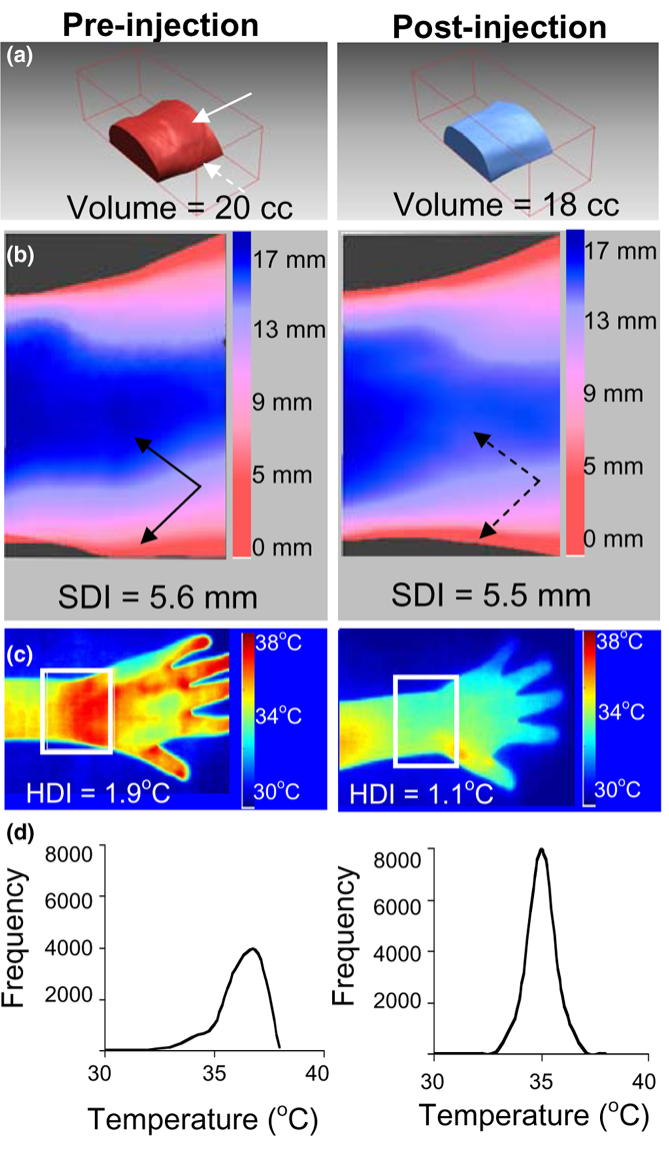
Changes in three-dimensional and thermal measurements after intra-articular steroid injection. A 9-year-old female with polyarticular juvenile idiopathic arthritis underwent imaging before and 5 days after an intra-articular steroid injection of the left wrist. **(a) **Pre- and post-injection wrist region of interest and volume with dorsal (solid white arrow) and lateral (dashed white arrow) swelling evident in the pre-injection image. **(b) **Pre- and post-injection surface distance color map demonstrating pre-injection swelling (solid arrows) that resolves post-injection (dashed arrows). **(c) **Pre- and post-injection thermograms and heat distribution index (HDI). **(d) **Pre- and post-injection relative frequency distributions of temperatures. SDI, surface distribution index.

To demonstrate the potential utility of 3D and thermal surface imaging to monitor arthritis, we have longitudinally imaged two patients with wrist arthritis. The first patient was a 9-year-old female with anti-nuclear antigen (ANA)-negative and rheumatoid factor (RF)-negative polyarticular JIA who presented with left wrist pain, warmth, and swelling. The decision was made to proceed with intra-articular steroid injection, and the patient underwent imaging prior to the procedure (Figure 4). The patient returned for re-imaging 5 days after the injection. A reduction in volume of 2 ml, representing a 10% decrease, was noted (Figure 4a). No significant change in SDI was observed, although the area of decreased swelling was evident on the surface color map (Figure 4b). HDI values changed from 1.9°C prior to the injection to 1.1°C after the injection (Figure 4c), associated with narrowing of her temperature frequency distribution (Figure 4d). These quantitative findings correlated with both physician-assessed improvement in swelling and tenderness and patient report of symptom reduction.

**Figure 5:**
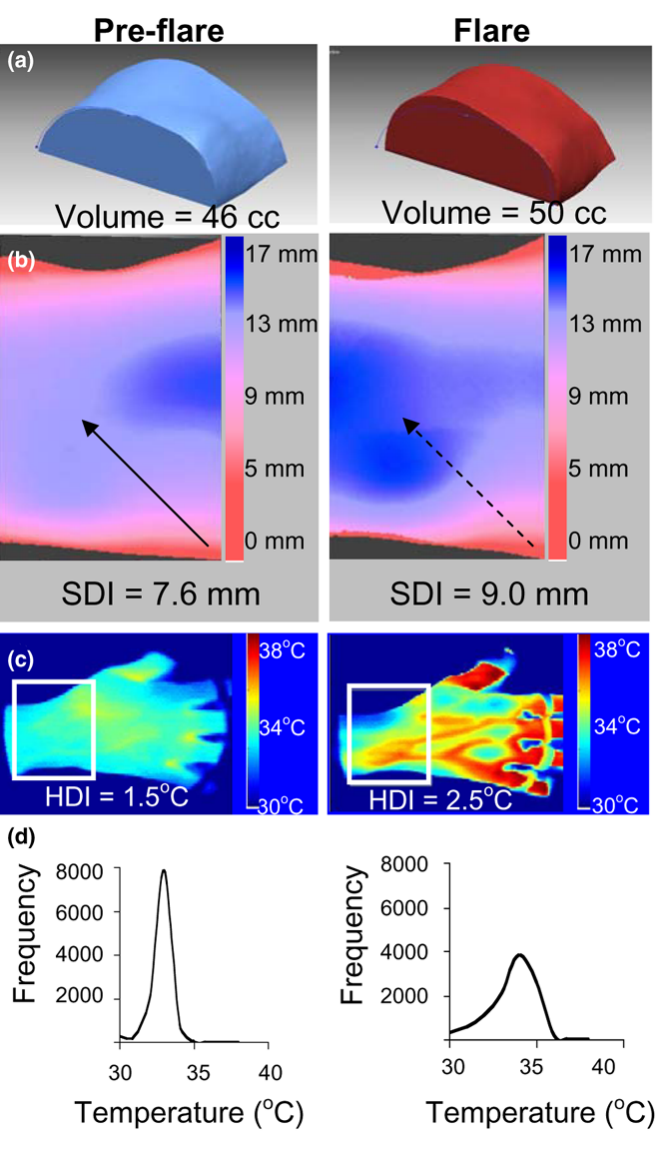
Changes in three-dimensional and thermal measures during rheumatoid arthritis flare. A 45-year-old Caucasian female with well-controlled rheumatoid arthritis was imaged. Nine days later, she developed an acute flare of her symptoms and was re-imaged on day 10. **(a) **Pre- and post-flare wrist region of interest and volume. **(b) **Pre- and post-flare surface distance color map demonstrating post-flare swelling (dashed arrow), not present in pre-flare image (solid arrow). **(c) **Pre- and post-flare thermograms and heat distribution index (HDI). **(d) **Pre- and post-flare relative frequency distributions of temperatures. SDI, surface distribution index.

The second patient was a 45-year-old Caucasian female with long-standing RF-positive RA on a regimen of hydroxychloroquine and methotrexate. She underwent imaging after completing a course of oral steroids for flare of her disease. At this initial imaging session, her symptoms and physical exam findings were minimal. Ten days later, she returned with complaints of increased swelling, stiffness, pain, and warmth in the right wrist. Her wrist was re-imaged. An increase in swelling, particularly on the dorsolateral aspect of the wrist, was visually apparent in the 3D models. Wrist volume increased between sessions by 4 ml, representing an 8.7% increase from baseline (Figure 5a). Wrist SDI increased between sessions by 1.4 mm, representing an 18.4% increase, along with an obvious change in surface contour as reflected by the surface color map (Figure 5b). The patient's wrist HDI increased from 1.5°C to 2.5°C (Figure 5c). Relative frequency distribution went from narrow to broad (Figure 5d). These quantitative findings correlated with both physician assessment of disease activity and patient report of worsening symptoms.

## Discussion

The findings from this proof-of-concept study suggest that surface imaging could be used to improve the assessment of disease activity in arthritis. Although the number of subjects we analyzed was small and will require further validation, our results demonstrate that this approach is feasible. The 3D measures described in this study were accurate and sensitive to small changes in joint volume and shape. HDI values of greater than 1.3°C could be used to identify patients with active arthritis. In 2 arthritis patients with changes in clinical status, these surface imaging measures were able to quantify changes that correlated with subjective physician assessment.

Currently used measures to monitor changes in arthritis activity, such as the ACR 20 and ACR 30, rely upon the number of joints with active arthritis as a core criterion [1,2]. However, multiple studies have documented the limited reproducibility of rheumatologist-assessed active or swollen joint counts. The inter-observer agreement of active joint count ranges from 0.69 to 0.76 [4,14]. Guzmán and colleagues [4] reported poor inter-rater agreement in the assessment of active disease in the wrist and MCPs. Similarly, in a study of patients with psoriatic arthritis, the inter-rater agreement regarding the number of swollen joints was even lower (ICC 0.10) [14]. Slightly higher agreement between observers in the assessment of swollen joints has been observed in other studies, with ICCs ranging from 0.7 to 0.82 [3,5,15]. ICCs reported in our study for 3D volume and SDI measures of the wrist and MCP were all greater than 0.99, a substantial improvement in reliability. Thus, surface imaging could improve the reliability of the active or swollen joint counts, which would lead to an overall improvement in the reliability of the ACR 20 and ACR 30.

We used a non-contact 3D laser scanning device used by other investigators to obtain objective and quantifiable data of the physical characteristics of body surfaces in non-arthritic conditions [16-21]. Highton and colleagues [22,23] used static laser technology to assist examiners in determining changes in joint size and hand function resulting from arthritis. This method required examiners to adjust the position of a laser beam on a joint surface and then record its position as a way to measure joint deformity. While this was a significant step toward objectifying shape changes in arthritis, there was still the potential for inter- and intra-user variability and only limited areas of the joints were examined. Our technology differed in that we examined the entire dorsal surface of the joint and data were acquired and recorded without user input, thus reducing the chance for operator variability.

Infrared thermography has been studied since the 1960s to measure active arthritis with variable results [24-37]. Multiple indices have been developed to quantify the temperature changes observed in arthritis [35,38]. The HDI measure used in our study reduces the environmental effects on absolute skin temperature [32]. Previous studies demonstrated that HDI, calculated by limiting the data to values greater than 15% of the modal frequency, correlated with the Ritchie articular index, grip strength, morning stiffness, erythrocyte sedimentation rate, and pain score [33]. In our study, the HDI performed with greater sensitivity when the data were not limited by modal frequency. Using thermal imaging, we determined that an HDI of greater than 1.3°C correlated with physician-assessed active arthritis (*r *= 0.68, *p *< 0.0001) and displayed a specificity of 100% and a sensitivity of 67% when compared with normal controls. The poorer performance of the MCP HDI is likely a consequence of uncontrollable physiologic factors (metabolic rate, caloric intake, and so on) within each subject, suggesting that absolute changes in HDI may not be a reliable longitudinal measure of change in arthritis activity. However, the HDI could be employed in a dichotomous fashion to classify joints as active or inactive, which could simplify and improve the reproducibility of active joint counts.

Other imaging modalities, such as MRI and ultrasound, have been proposed as tools to improve reproducibility and quantify changes in arthritic joints. Unlike 3D and thermal surface imaging, which collect exterior joint data, these other modalities examine structures below the joint surface. MRI has been used to quantify synovial volumes in JIA and RA [3,39]. Using the Rheumatoid Arthritis Magnetic Resonance Imaging Scores (RAMRIS), researchers have been able to document intra- and inter-rater correlation coefficients of greater than 0.89 in the assessment of synovitis [40,41]. However, MRI-measured synovial volumes require contrast administration and are time-intensive, requiring acquisition times of more than 20 minutes per extremity, and slightly less time to analyze the images [3]. In this study, using manual image acquisition and processing, patient positioning and image acquisition were completed in less than 5 minutes and image processing was completed in less than 30 minutes. These steps are amenable to full automation, which should result in a much shorter interval between imaging and availability of results.

Several previous studies have reported ultrasound's increased sensitivity in the detection of synovitis when compared with clinical assessment [40,42]. Naredo and colleagues [43] compared ultrasound to physician assessment of joint activity. Ultrasound exhibited greater reliability and sensitivity in the detection of synovitis and effusion compared with clinical examination. However, ultrasound is ultimately reliant on consistent performance by the operator. The same study reported moderate intra-observer agreement of ultrasound-measured effusions in the wrist and MCPs (kappa statistic 0.59 and 0.83, respectively) and synovitis in the wrist and MCPs (kappa statistic 0.62 and 0.76, respectively).

For this study, pediatric arthritis and adult arthritis were considered as a single group since the study was designed (a) to determine the ability of the thermal and 3D cameras to provide reproducible data from repeated imaging of the same wrist and (b) to detect a difference between wrists with arthritis and control wrists. Therefore, the adult and pediatric arthritis subjects were considered as a single group representing wrists with inflammation and compared with a single control group. Analyzed in this manner, the number of subjects was adequate for the study, as demonstrated by the very significant *p *values. In the future, it would be of interest to study JRA separately to see whether very small children would be able to cooperate with the imaging protocol.

In our study, novel 3D and thermal surface imaging techniques and post-processing methods were developed and tested in a clinically relevant setting. The wrist and 2nd-5th MCPs were selected as targets over other joints given their frequent involvement in RA and JIA. Since this was a proof-of-concept study aimed at establishing the ability of surface imaging technologies to quantify physical changes of arthritis, other small joints such as the 1st MCP and proximal interphalangeals were not examined. However, techniques developed in this study can be easily adapted for use in the assessment of any other peripheral joint. In addition, imaging was performed only on the dorsal half of these joints since this is the primary surface evaluated clinically by the rheumatologist and allows the use of a simple fixation splint and to limit the scans necessary to provide coverage of the ROI to two per model.

To follow patients longitudinally, it was essential to prevent minor wrist or hand rotation from session to session which might cause false variations in volume measurements. The fixation device constructed for this study prevented most such rotation. Small positioning changes that did occur were readily overcome by aligning the forearm and hand of models created across sessions, using specially developed co-registration functions. Furthermore, the virtual 3D ROI boxes we created are of fixed size sufficient to allow for progressive shape changes over time. However, it is possible that, in severe deformity, additional measures may be needed to image the entire region. For example, we have found that an additional 3D view taken from the anterior aspect of the hand allows us to capture the surface of very deformed MCP joints. While further optimization of the fixation device may be necessary in order to ensure reproducible positioning between clinic visits, the innovative methods and technologies developed during our study may someday result in a clinical device that provides a rapid and accurate longitudinal assessment of disease activity.

## Conclusion

In the present study, we have established the ability of 3D and thermal surface imaging to produce reliable, quantifiable measures of joint volume, shape, and temperature to aid in the assessment of disease activity in arthritis. We are currently assessing the inter-observer reliability and the effect of significant deformity on this approach in a larger population of RA and JIA patients. Ultimately, this approach may provide a tool to improve the accuracy of assessment of arthritis.

## Abbreviations

3D = three-dimensional; ACR = American College of Rheumatology; CV = coefficient of variation; HDI = heat distribution index; ICC = intra-class correlation coefficient; JIA = juvenile idiopathic arthritis; MCP = metacarpalphalangeal; MRI = magnetic resonance imaging; RA = rheumatoid arthritis; RF = rheumatoid factor; ROC = receiver operating characteristic; ROI = region of interest; SD = standard deviation; SDI = surface distribution index.

## Competing interests

LD, RH, DH, and CKK have equity interest in Cartesia Dx (Pittsburgh, PA, USA). SJS, RB, JE, and JL declare that they have no competing interests.

## Authors' contributions

SJS participated in study design, data acquisition, processing, analysis, and in preparation of the manuscript. JE participated in data acquisition and analysis. CKK participated in study design and helped to draft the manuscript. RB participated in the design of the study and performed the statistical analysis. JL constructed the fixation device and participated in study design. DH and LD helped with study design. RH conceived of the study, participated in its design and coordination, and helped to draft the manuscript. All authors read and approved the final manuscript.
